# Four new troglophilic species of *Loxosceles* Heinecken & Lowe, 1832: contributions to the knowledge of recluse spiders from Brazilian caves (Araneae, Sicariidae)

**DOI:** 10.3897/zookeys.806.27404

**Published:** 2018-12-13

**Authors:** Rogério Bertani, Diego M. von Schimonsky, Jonas E. Gallão, Maria E. Bichuette

**Affiliations:** 1 Laboratório Especial de Ecologia e Evolução, Instituto Butantan, Av. Vital Brasil, 1500 CEP 05503-900, São Paulo, São Paulo, Brazil; 2 Laboratório de Estudos Subterrâneos, Departamento de Ecologia e Biologia Evolutiva, Universidade Federal de São Carlos campus São Carlos, São Paulo, Brazil; 3 Programa de Pós Graduação em Biologia Comparada – Faculdade de Filosofia, Ciências e Letras de Ribeirão Preto - FFCLRP, Universidade de São Paulo – USP, Ribeirão Preto, São Paulo, Brazil

**Keywords:** Bahia, brown spider, karst area, Minas Gerais, taxonomy

## Abstract

Four new species of recluse spiders from Brazilian caves are described with both males and females. *Loxoscelesericsoni* Bertani, von Schimonsky & Gallão, **sp. n.** and *L.karstica* Bertani, von Schimonsky & Gallão, **sp. n.** both occur in caves in the Peruaçu region, located in the northern area of the state of Minas Gerais; *L.karstica***sp. n.** is additionally found in the Serra do Ramalho karst area, located in the southwestern region of the state of Bahia. These two species belong to the *gaucho* group. *Loxoscelescarinhanha* Bertani, von Schimonsky & Gallão, **sp. n.** and *L.cardosoi* Bertani, von Schimonsky & Gallão, **sp. n.** occur exclusively in caves of the Serra do Ramalho karst area and belong to the *rufescens*/*amazonica* species group. The discovery of two additional and highly distinct species in the *rufescens*/*amazonica* group (*L.carinhanha***sp. n.** and *L.cardosoi***sp. n.**) increases the debate on the origin, evolution, and geographical distribution of this widely distributed group of recluse spiders in the New and Old World. The presence of three species (*L.ericsoni***sp. n.**, *L.carinhanha***sp. n.**, and *L.cardosoi***sp. n.**) with marked differences in morphological characters in a relatively small area indicates that the region seems to be an important center for *Loxosceles* diversity, which remains poorly studied.

## Introduction

The genus *Loxosceles* Heinecken & Lowe, 1832, known as brown or recluse spiders, comprises 134 species (World Spider Catalog 2018) from the New World, Africa, Europe, and Asia. Many species are of medical concern due to the potent venom they produce, which can cause severe necrosis following a bite (Gertsch 1967, Isbister and Fan 2011). Most of the species found in the New World were described by Gertsch and Mulaik (1940), Gertsch (1958, 1967, 1973), and Gertsch and Ennik (1983). After these significant revisions, very few species were described (Wang 1994, Martins et al. 2002), but more recently, *Loxosceles* is again receiving attention with several new species described (Ribera and Planas 2009, Duncan et al. 2010, Bertani et al. 2010, González-Sponga 2010, Gonçalves-de-Andrade et al. 2012, Sánchez-Ruiz and Brescovit 2013, Planas and Ribera 2015, Cala-Riquelme et al. 2015, Fukushima et al. 2017, Tahami et al. 2017; Lotz 2017; Brescovit et al. 2017; Souza and Ferreira 2018).

In South America, Gertsch (1967) distinguished four groups of species, *gaucho*, *laeta*, *spadicea*, and *amazonica*. The *gaucho* group now has six species in Brazil, as follows: *L.gaucho* Gertsch, 1967; *L.adelaida* Gertsch, 1967; *L.similis* Moenkhaus, 1898; *L.chapadensis* Bertani, Fukushima & Nagahama, 2010; *L.niedeguidonae* Gonçalves-de-Andrade, Bertani, Nagahama & Barbosa, 2012; *L.troglobia* Souza & Ferreira, 2018, and one species from Paraguay, *L.variegata* Simon, 1897. The *spadicea* group has three species recorded in Brazil: *L.intermedia* Mello-Leitão, 1934; *L.hirsuta* Mello-Leitão ,1931; and *L.anomala* (Mello-Leitão, 1917); and one species in Bolivia, *L.spadicea* Simon, 1907. The *laeta* group is the largest, with 24 species described by Gertsch (1967). A single native species was described in Brazil, *L.puortoi* Martins, Knysak & Bertani, 2002, and one was introduced, *L.laeta* (Nicolet, 1849). It is especially diverse in the Peruvian Andes. Gertsch (1967) considered the *amazonica* group to have a single species, *L.amazonica* Gertsch, 1967, from Brazil. Recently, it was proposed that this species belongs to an Old World group, *rufescens* (Fukushima et al. 2017), and two additional species from Brazil were described: *L.williansoni* Fukushima, Gonçalves-de-Andrade & Bertani, 2017 and *L.muriciensis* Fukushima, Gonçalves-de-Andrade & Bertani, 2017 (Fukushima et al. 2017). Phylogenetic analyses using molecular (Binford et al. 2008, Duncan et al. 2010) or morphological approach (Magalhães et al. 2017) were recently published, but they included a limited subset of *Loxosceles* species, or focused mainly on the *rufescens* group (Binford et al. 2008, Duncan et al. 2010).

*Loxosceles* are secretive spiders found under rocks, ground litter, and loose bark; in the holes of trees, tree trunks, and natural openings in cliffs and banks; and in caves (Gertsch 1967). The majority of *Loxosceles* found in caves are troglophiles, which means that they have source populations both inside and outside caves, completing its life cycle in both environments, however, troglobitic species (obligatory and exclusive subterranean source population) may be found (*sensu*Trajano 2012). There are 15 described species of *Loxosceles* in Brazil, and only four, *L.adelaida*, *L.similis*, *L.willianilsoni*, and *L.troglobia* (the only troglobitic species in Brazil), occur in caves. However, it is worth mentioning that the majority of the records of *Loxosceles* in Brazilian caves are still at a generic level (Trajano 1987, Pinto-da-Rocha 1995, Cordeiro et al. 2014, Gallão and Bichuette 2015). Worldwide, this genus has been recorded in caves in Iran, Thailand, South Africa, and Namibia (e.g., Chomphuphuang et al. 2016, Tahami et al. 2017, Lotz 2017). It is also noteworthy that, for some caves in Namibia and South Africa, there are records of at least seven species, two of them coexisting in one cave in South Africa (*L.parramae* Newlands, 1981 and *L.speluncarum* Simon, 1893) (Lotz 2017).

The aim of this paper is to describe four new *Loxosceles* species with a discussion about distribution and diversity of this genus in Brazilian caves.

## Materials and methods

### Study sites

Studied regions are in the transition zone of the Cerrado and Caatinga morphoclimatic domains (Ab’Saber 1977), and, according to the Koppen-Geiger classification (Peel et al. 2007), the climate is tropical semi-arid, with a well-defined dry period between April and September and an average annual temperature of 24 °C and a maximum rainfall of 800–1000 mm (INMET 2017).

*Peruaçu region, in the northern area of the state of Minas Gerais in southeastern Brazil*

**Figure 1.**
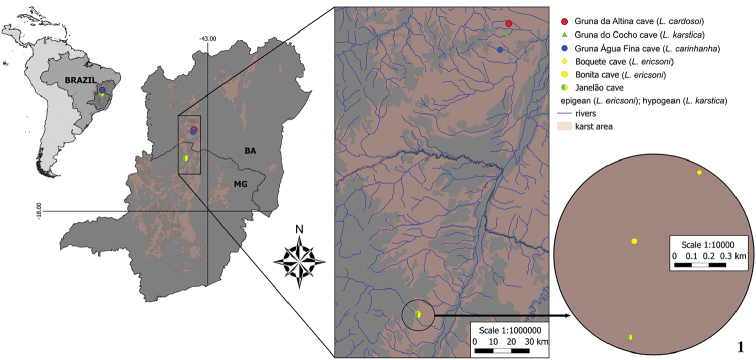
Map showing records of *L.ericsoni* sp. n., *L.karstica* sp. n., *L.carinhanha* sp. n., and *L.cardosoi* sp. n

**Figures 2–6.**
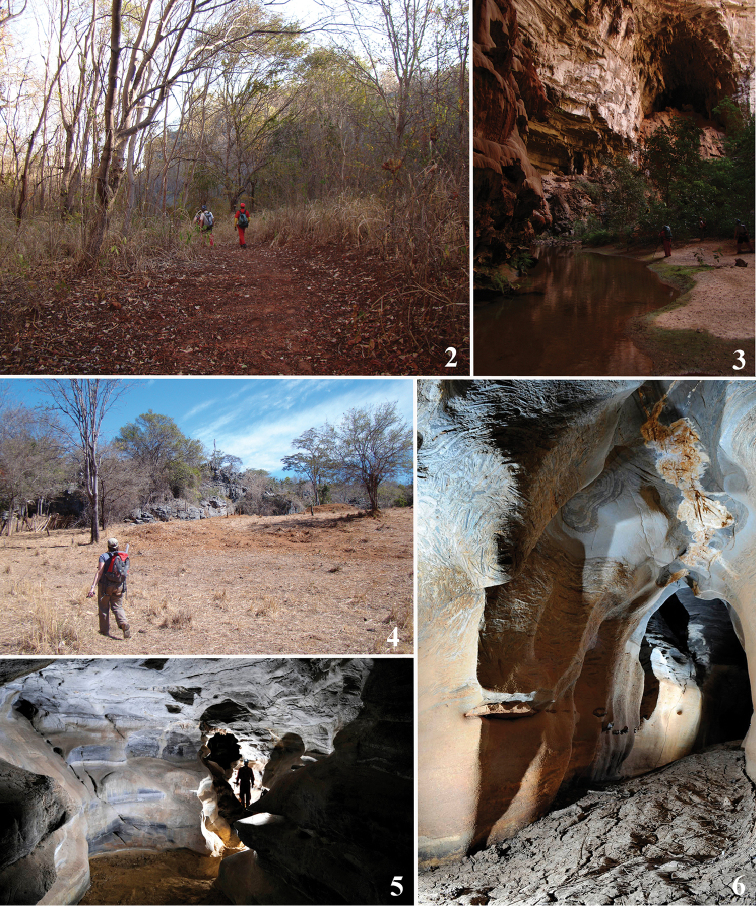
Habitats. **2** Caatinga vegetation at Peruaçu Caves National Park, Januária, state of Minas Gerais, Brazil **3** Janelão Cave **4** Caatinga vegetation at Serra do Ramalho karst area, Carinhanha, state of Bahia, Brazil **5** Gruna da Altina Cave **6** Gruna Água Fina Cave. Photographs by PP Rizzato (**2–4**), A Gambarini (**5, 6**).

The Janelão, Bonita, and Boquete Caves (Figs 1–3) are located in Peruaçu Caves National Park (PCNP), in the state of Minas Gerais in southeastern Brazil, and are under legal protection. The region is covered by extensive limestone outcrops of the Bambuí geomorphological group (Piló and Kohler 1991) and is home to the richest cave in Minas Gerais, Olhos d’Água Cave, with at least 12 obligatory cave species of troglobites (Trajano et al. 2016, Gallão and Bichuette 2018).

*Serra do Ramalho karst area, state of Bahia, northeastern Brazil*

The Serra do Ramalho karst area (Figs 1, 4–6) comprises several limestone outcrops of the Bambuí geomorphological group, including several large cave systems, some reaching several kilometers in length (Auler et al. 2001). The region is one of high subterranean diversity, and there is no legal protection for this region (Trajano et al. 2016). The main threats are deforestation for agriculture and pastureland, in addition to potential mining projects for cement production and mineral products (Gallão and Bichuette 2018).

### Specimens

Gertsch (1967) was used as the basis for species descriptions. Structures from the left side of the specimens were used, or, when the right side was used, the figures were mirrored to show them as coming from the left side to allow for easy comparison. A Leica LAS Montage and LAS 3D module mounted on a Leica M205C dissecting microscope were used for image capture and measurements of spider structures. Left legs and palps were measured from the dorsal aspect of the left side. All measurements are in millimeters. The copulatory organs of females were dissected and digested with a commercial protein remover for contact lenses (with pancreatin) for several minutes in order to observe the internal structure; when necessary, they were also cleared with clove oil.

**Abbreviations**:

**ALE** anterior lateral eye,

**PLE** posterior lateral eye,

**PME** posterior median eye.

Most specimens were collected inside caves and fixed with ethanol 70%. Epigean collections were conducted in the cave surroundings. Specimens are deposited at **LES** – Laboratório de Estudos Subterrâneos, Universidade Federal de São Carlos, São Carlos (curator ME Bichuette); and at **MZUSP** – Museu de Zoologia da Universidade de São Paulo (USP), São Paulo (curator R Pinto-da-Rocha).

## Taxonomy

### Loxosceles ericsoni

Taxon classificationAnimaliaAraneaeSicariidae

Bertani, von Schimonsky & Gallão sp. n.

http://zoobank.org/E39A83F9-474A-4657-ABB8-5F914EA6416F

Figs 1 , 7–12 , 13–17 , 55

#### Type material.

Male holotype (MZUSP 74427) and female paratype (MZUSP 74429), BRAZIL: *Minas Gerais*, Januária, Epigean Janelão Cave (15°06'S, 44°14'W) 600 m a.s.l., M.E. Bichuette, P.P. Rizzato, and J.E. Gallão leg., 22.vii.2012; Boquete Cave (15°04'S, 44°17'W) 681 m a.s.l., 1 female paratype, same collectors and date (MZUSP 74430); Bonita Cave (15°06'S, 44°14'W) 661 m a.s.l., paratypes 4 females, same collectors and date (LES 14592).

#### Other material examined.

BRAZIL, *Minas Gerais*: Januária, epigean habitats near Janelão Cave (15°06'S, 44°14'W) 600 m a.s.l., 2 immatures, M.E. Bichuette, P.P. Rizzato and J.E. Gallão leg., 22.vii.2012 (MZUSP 74428); Boquete Cave, 681 m a.s.l., 1 female, 2 immatures, M.E. Bichuette, P.P. Rizzato, and J.E. Gallão leg., 23.vii.2012 (LES 14593), 2 immatures, same collectors and date (MZUSP 74431); Bonita Cave, 681 m a.s.l., 3 immatures, same collectors and date (LES 14594).

#### Diagnosis.

Males of *Loxoscelesericsoni* sp. n. resemble those of *L.karstica* sp. n. by the palpal tibia length being less than 2 and more than 1.4 times the palpal cymbium length. They can be distinguished from *L.karstica* sp. n. by the longer cymbium and slender embolus (Figs 11, 12). Females of *L.ericsoni* sp. n. differ from those of all other *Loxosceles* species by the extremely narrow sclerotized transversal plate and the two straight, very long, and slender receptacles (Figs 15–17).

**Figures 7–12.**
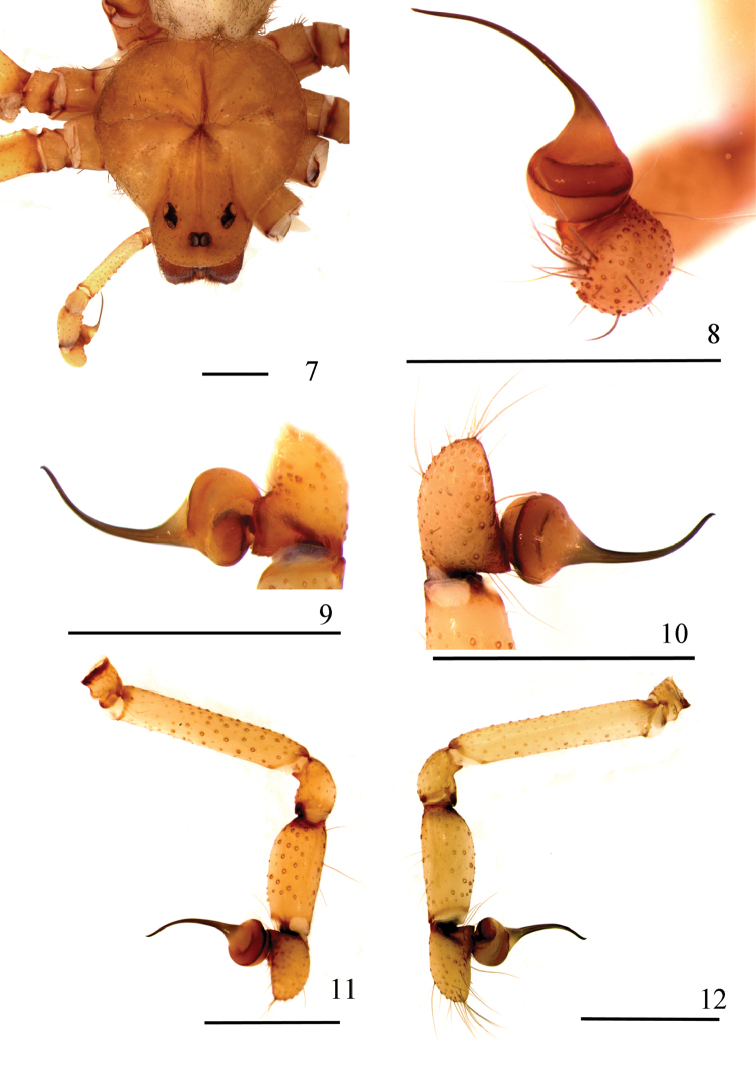
*Loxoscelesericsoni* sp. n., holotype male (MZUSP 74427) **7** carapace and palp **8−10** left palpal bulb **8** dorsal **9** retrolateral **10** prolateral **11, 12** left palp **11** prolateral **12** retrolateral. Scale bar: 1 mm.

#### Description.

*Male holotype*: Total length 6.85. Carapace 3.20 long, 3.04 wide. Eye sizes and interdistances: ALE 0.23, PME 0.21, PLE 0.22, PME-PLE 0.04, PME-ALE 0.25; clypeus 0.35. Leg formula II, I, IV, III. Leg lengths: leg I: femur 8.73, patella 1.23, tibia 10.71, metatarsus 10.68, tarsus 1.93, total 33.28; II (right leg, left missing): 11.87, 1.27, 15.01, 15.80, 1.70, 45.65; III: 8.46, 1.15, 8.65, 11.22, 1.59, 31.07; IV: 9.29, 1.23, 9.73, 12.42, 1.80, 34.47. Palp: femur 1.53 long, 0.27 wide; patella 0.45 long, 0.32 wide; tibia 0.87 long, 0.35 wide; cymbium 0.62 long, 0.33 wide. Labium 0.63 long, 0.56 wide. Sternum 1.58 long, 1.60 wide. Femur I 2.7 times as long, tibia I 3.3 times as long and leg I 10.4 as long as carapace. Palpal femur 5.6 times longer than wide, tibia 2.5 times longer than wide, cymbium longer than wide (Figs 11, 12). Bulb suboval and slightly longer than half cymbium length. Embolus slender, long, gently curved and ending in a short and steep curvature on apex, approximately 2.4 times longer than bulb length in retrolateral view, without carina (Figs 8–10). Cephalic region of carapace with some sparse long setae (Fig. 7). Carapace with light brown pars cephalica and border (Fig. 7). Legs and palp light brown, covered by short, greyish setae. Coxae, endites, and sternum light brown.

**Figures 13–17.**
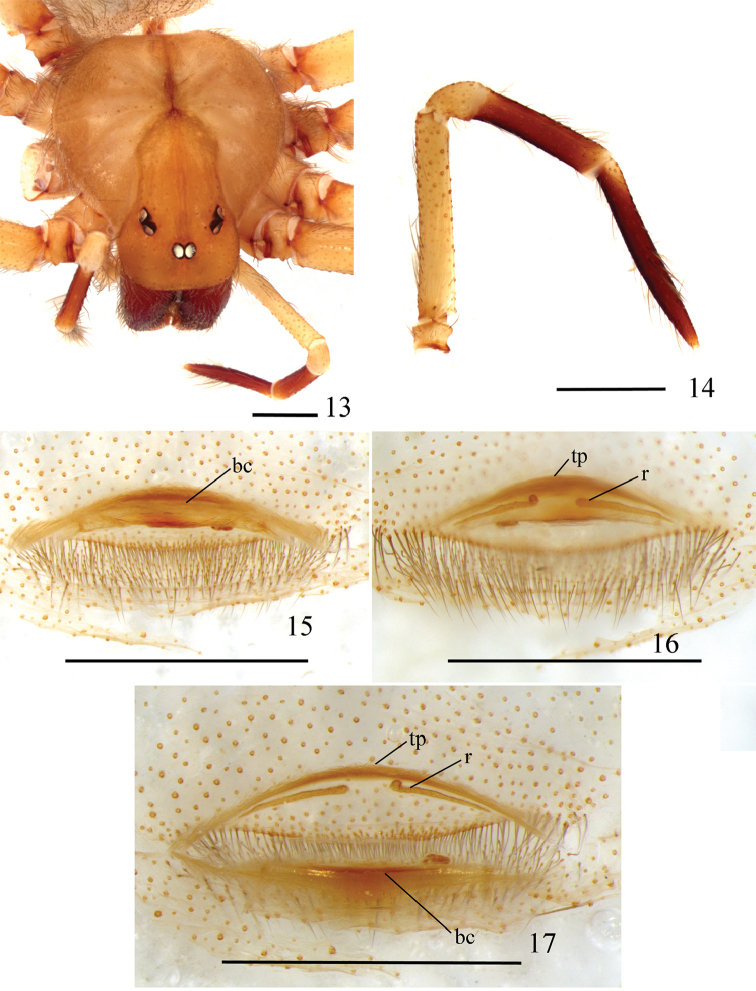
*Loxoscelesericsoni* sp. n., paratype female (MZUSP 74429) **13** carapace and palp **14** left palp, prolateral **15−17** spermathecae **15** dorsal, with bursa copulatrix over receptacles **16** ventral **17** dorsal, bursa copulatrix unfolded below. Abbreviations: bc bursa copulatrix, r receptacle, tp transverse sclerotized plate. Scale bar: 1 mm.

*Female paratype*: Total length 9.37. Carapace 4.04 long, 3.55 wide. Eye sizes and interdistances: ALE 0.26, PME 0.21, PLE 0.24, PME-PLE 0.01, PME-ALE 0.56; clypeus 0.42. Leg formula II, I, IV, III. Leg lengths: leg I: femur 8.41, patella 1.42, tibia 10.05, metatarsus 9.41, tarsus 1.87, total 31.16; II: 9.70, 1.32, 11.66, 12.34, 1.92, 36.94; III: 7.98, 1.33, 7.58, 9.35, 1.51, 27.75; IV: 8.48, 1.34, 8.64, 10.98, 1.89, 31.33. Palp: femur 2.05 long, 0.35 wide; patella 0.58 long, 0.38 wide; tibia 1.27 long, 0.26 wide; tarsus 1.94 long, 0.20 wide. Labium 0.69 long, 0.66 wide. Sternum 2.18 long, 1.96 wide. Femur I 2.1 times as long, tibia I 2.5 times as long, and leg I 7.7 as long as carapace. Palpal femur 5.8 times longer than wide, tibia 4.9 longer than wide, tarsus not incrassate (Fig. 14). Spermathecae sclerotized transverse plate extremely narrow. Two receptacles almost straight, long, slender, parallel to transverse sclerotized plate (Figs 15–17). Dorsal part of the bursa copulatrix with a central area medially sclerotized (Fig. 17). Cephalic region of carapace with some sparse long setae (Fig. 13). Carapace with light brown pars cephalica and border (Fig. 13). Legs light brown, covered by short, greyish setae. Palp light brown, except reddish brown tibia and tarsus (Fig. 14). Coxae and sternum light brown. Endites and labium brown.

#### Etymology.

The specific name is in honor of Ericson Cernawsky Igual from Grupo Pierre Martin de Espeleologia (GPME) for his contribution to Brazilian speleology and his commitment to the conservation of caves.

#### Remarks.

*Loxoscelesericsoni* sp. n. females have highly modified spermathecae (Figs 15–17). Although they have a transverse plate, it is very narrow and does not connect directly to the two receptacles (Figs 16, 17). The receptacles themselves are two long, slender tubes positioned parallel to the transverse plate and converging to the center. Despite the modified female genitalia, it is possible to include this species in the *gaucho* group by the male palpal morphology, as they have a cymbium almost the same length as the palpal tibia (Figs 11, 12).

### Loxosceles karstica

Taxon classificationAnimaliaAraneaeSicariidae

Bertani, von Schimonsky & Gallão sp. n.

http://zoobank.org/1E1BBD45-081B-44DF-9ED1-DC1F1581F466

Figs 1 , 18–21 , 22–27

#### Material examined.

Female holotype (MZUSP 74432), male paratype (MZUSP 74433), 2 female paratypes (LES 14712), 2 male and 2 female paratypes (MZUSP 74434), 3 female paratypes (MZUSP 74435), 3 male and 3 female paratypes (LES 14595), BRAZIL: *Minas Gerais*, Januária, Janelão Cave (15°06'S, 44°14'W) 600 m a.s.l., M.E. Bichuette, P.P. Rizzato and J.E. Gallão leg., 22.vii.2012.

#### Other material examined.

BRAZIL, *Minas Gerais*: Januária, Janelão Cave (15°06'S, 44°14'W) 600 m a.s.l., 2 immatures, M.E. Bichuette, P.P. Rizzato and J.E. Gallão leg., 22.vii.2012 (LES 14596), 1 immature, same collectors and date (LES 14713); *Bahia*: Carinhanha, Gruna do Cocho Cave (13°36'S, 43°46'W) 514 m a.s.l., 3 females, M.E. Bichuette, N. Hattori and J.E. Gallão leg., 02.vi.2012 (LES 14597).

#### Diagnosis.

Males of *Loxosceleskarstica* sp. n. resemble those of *L.ericsoni* sp. n. by the palpal tibia length more than 1.4 and less than 2.0 times the cymbium length. They can be distinguished from those of *L.ericsoni* sp. n. by the shorter cymbium and stouter embolus (Figs 26, 27). Females of *L.karstica* sp. n. resemble those of *L.similis*, *L.chapadensis*, and *L.niedeguidonae* by the spermathecae having the sclerotized transversal plate with two long and straight receptacles. They differ from the females of the species above by the short, sclerotized transverse plate with the receptacles positioned at an angle of 45° to the inner side (Figs 20, 21). Additionally, they can be distinguished from females of *L.chapadensis* by the dorsal part of the bursa copulatrix medially sclerotized (Fig. 21) and from *L.niedeguidonae* by the non-incrassated palpal tarsus (Fig. 19).

**Figures 18–21.**
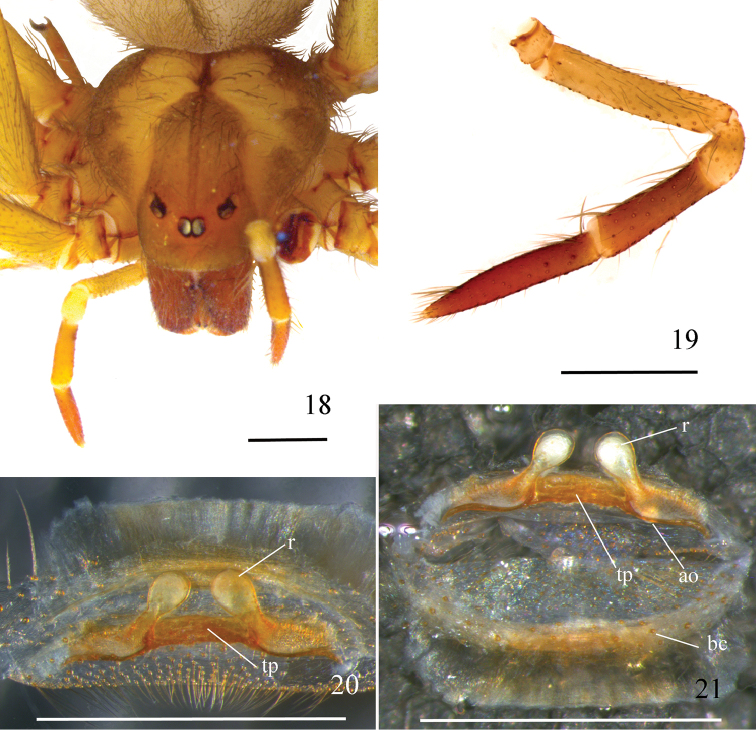
*Loxosceleskarstica* sp. n., holotype female (MZUSP 74432) **18** carapace and palp **19** left palp, prolateral **20, 21** spermathecae **20** ventral **21** dorsal, bursa copulatrix unfolded below. Abbreviations: ao atriobursal orifice, bc bursa copulatrix, r receptacle, tp transverse sclerotized plate. Scale bar: 1 mm.

#### Description.

*Female holotype*: Total length 8.60. Carapace 3.58 long, 3.16 wide. Eye sizes and interdistances: ALE 0.24, PME 0.21, PLE 0.24, PME-PLE 0.05, PME-ALE 0.20; clypeus 0.32. Leg formula II, I, IV, III. Legs length: leg I: femur 7.49, patella 1.18, tibia 8.10, metatarsus 7.79, tarsus 1.79, total 26.35; II: 8.29, 1.25, 9.01, 9.05, 1.74, 29.34; III: 6.39, 1.28, 6.32, 7.17, 1.53, 22.69; IV: 7.41, 1.31, 7.43, 8.42, 1.58, 26.15. Palp: femur 1.44 long, 0.24 wide; patella 0.52 long, 0.34 wide; tibia 1.02 long, 0.29 wide; tarsus 1.41 long, 0.24 wide. Labium 0.80 long, 0.58 wide. Sternum 1.90 long, 1.74 wide. Femur I 2.1 times as long, tibia I 2.2 times as long, and leg I 7.3 as long as carapace. Palpal femur 6 times longer than wide; tibia 3.5 longer than wide; tarsus not incrassate (Fig. 19). Spermathecae sclerotized transverse plate short with almost-straight receptacles positioned at an angle of 45° to the inner side (Figs 20, 21). Dorsal part of the bursa copulatrix medially sclerotized (Fig. 21). Cephalic region of carapace with some sparse, long setae (Fig. 18). Carapace with dark-brown pars cephalica and border (Fig. 18). Legs brown, covered by short, greyish setae. Palp light brown, except for reddish brown tibia and tarsus (Fig. 19). Coxae and sternum light brown. Endites and labium brown.

**Figures 22–27.**
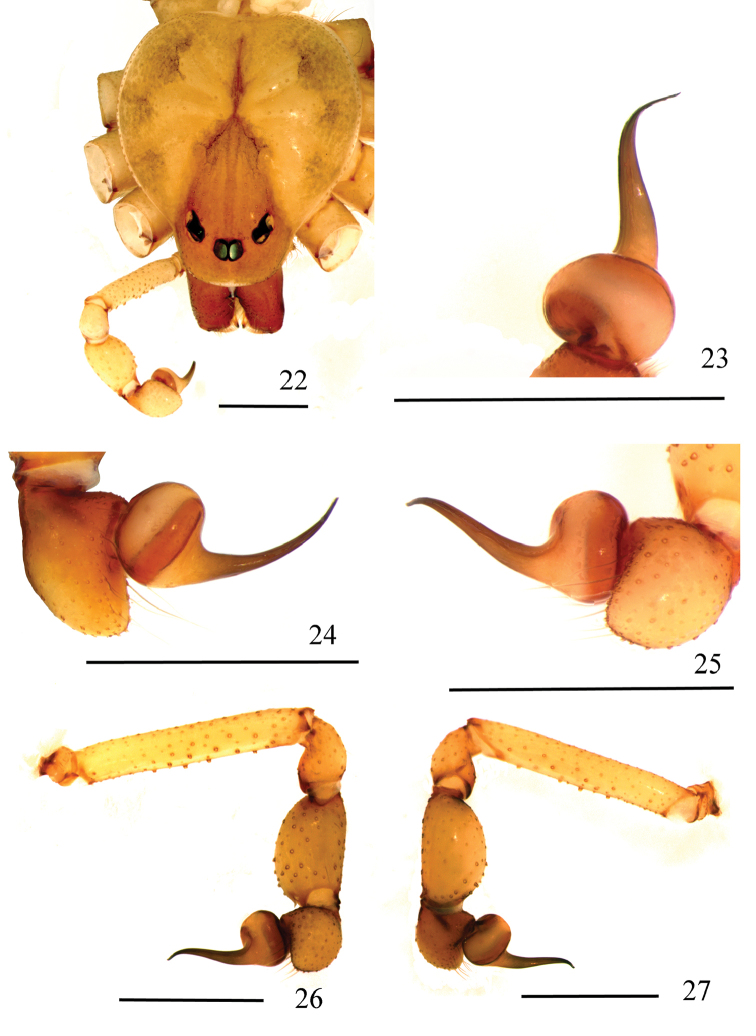
*Loxosceleskarstica* sp. n., paratype male (MZUSP 74433) **22** carapace and palp **23−25** left palpal bulb **23** dorsal **24** prolateral **25** retrolateral **26, 27** left palp **26** prolateral **27** retrolateral. Scale bar: 1 mm.

*Male paratype*: Total length 6.56. Carapace 3.06 long, 2.76 wide. Eye sizes and interdistances: ALE 0.26, PME 0.21, PLE 0.20, PME-PLE 0.04, PME-ALE 0.17; clypeus 0.32. Leg formula II, I, IV, III (inferred from male MZUSP 74434; see below). Leg lengths: leg I: missing; II: femur 9.39, patella 1.22, tibia 10.58, metatarsus 11.69, tarsus 0.92, total 33.80; III: missing; IV: 7.91. 1.24. 8.06. 9.50. 1.64. 28.35. Palp: femur 1.72 long, 0.29 wide; patella 0.51 long, 0.35 wide; tibia 0.84 long, 0.48 wide; cymbium 0.57 long, 0.44 wide. Labium 0.52 long, 0.44 wide. Sternum 1.67 long, 1.57 wide. Femur I 2.6 times as long, tibia I 3.1 times as long, and leg I 9.9 as long as carapace (inferred from male MZUSP 74434; see below). Palpal femur 5.9 times longer than wide, tibia 1.7 times longer than wide, cymbium oval (Figs 26, 27). Bulb suboval and a little smaller than cymbium. Embolus curved from its basis, approximately 1.8 times longer than bulb length in retrolateral view, without carina (Figs 23–25). Cephalic region of carapace covered by some sparse setae (Fig. 22). Entire pars cephalica dark-brown, carapace border dark-brown but slightly faded (Fig. 22). Legs, palps, endites, coxae, sternum, and labium light brown.

#### Remarks.

The male specimen in better condition to be chosen as paratype lacks legs I and III. For this reason, another male (MZUSP 74434), not in condition to serve as type, had legs measured as follows: leg I: femur 8.25, patella 1.16, tibia 9.88, metatarsus 10.33, tarsus 2.10, total 31.72; II: 9.89, 1.23, 11.72, 13.07, 2.02, 37.93; III: 7.49, 1.06, 7.51, metatarsus broken, tarsus missing; IV: 8.21, 1.06, 8.72, 10.24, tarsus missing. Carapace: 3.20 long, 2.94 wide.

The new species *L.karstica* sp. n. has genitalic characteristics shared with other species of the *gaucho* group distributed in the southern regions of Brazil and Paraguay, as *L.gaucho*, *L.similis*, *L.adelaida*, and *L.variegata*, which have palpal tibia that are short and incrassate in males (Figs 26, 27) and the large spermathecae transverse plate in females (Figs 20, 21). It also shares characteristics with the species of the *gaucho* group that are more distributed in the northern part of Brazil, as *L.chapadensis* and *L.niedeguidonae*, as they have a longer palpal tibia in males and a spermathecae transverse plate that is almost as short as in the new species but with differences in the bursa copulatrix sclerotization and the size and shape of receptacles.

#### Etymology.

The specific name refers to karst, a word used to define terrain with distinctive hydrology and landforms that arise from a combination of high rock solubility and well-developed porosity. *Loxosceleskarstica* sp. n. occurs in two important karst areas of Brazil (Peruaçu and Serra do Ramalho).

### Loxosceles carinhanha

Taxon classificationAnimaliaAraneaeSicariidae

Bertani, von Schimonsky & Gallão sp. n.

http://zoobank.org/F90FD902-CBA1-40A1-9B59-506D75D722D3

Figs 1 , 28–33 , 34–36 , 37–41

#### Material examined.

Male holotype (MZUSP 74436) and female paratype (MZUSP 74437), 1 female paratype (MZUSP 74438), 1 female paratype (LES 14709), BRAZIL: *Bahia*, Carinhanha, Gruna Água Fina cave (13°41'S, 43°48'W) 484 m a.s.l., M.E. Bichuette, N. Hattori and J.E. Gallão leg., 29.v.2012.

#### Other material examined.

BRAZIL, *Bahia*: Carinhanha, Gruna Água Fina Cave (13°41'S, 43°48'W) 484 m a.s.l., 1 female and 2 immatures, M.E. Bichuette, N. Hattori and J.E. Gallão leg., 29.v.2012 (MZUSP 74439).

#### Diagnosis.

Males of *Loxoscelescarinhanha* sp. n. can be distinguished from those of all other *Loxosceles* species by the thick embolus (Figs 29–31), a strong curvature on basal metatarsus I, and a constriction on distal tibia I (Figs 34, 35). Females of *L.carinhanha* sp. n. resemble females of *L.cardosoi* sp. n. by having spermathecae as a large, weakly sclerotized pouch with two large receptacles on its distal portion. Females of *L.carinhanha* sp. n. can be distinguished from those of *L.cardosoi* sp. n. by the spermathecae lacking a sclerotized transverse plate and dorsal parts of bursa copulatrix having only a small sclerotized triangular area (Figs 39–41).

**Figures 28–33.**
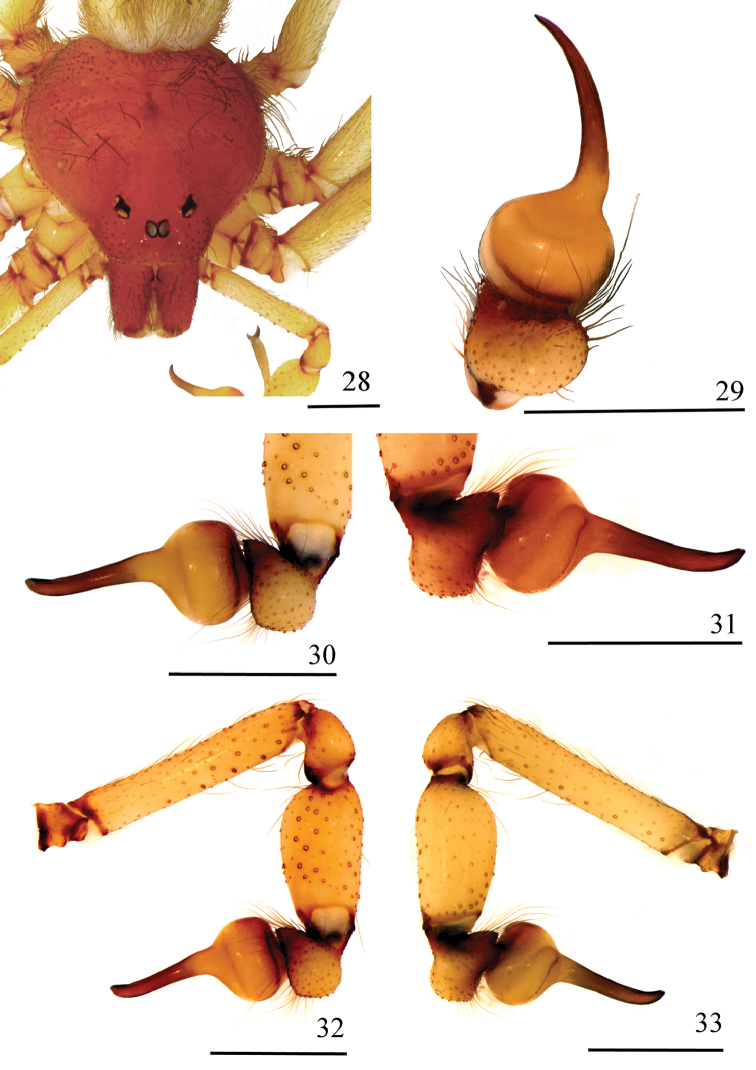
*Loxoscelescarinhanha* sp. n., holotype male (MZUSP 74436) **28** carapace and palp **29−31** left palpal bulb **29** dorsal **30** prolateral **31** retrolateral **32, 33** left palp **32** prolateral **33** retrolateral Scale bar: 1 mm.

#### Description.

**Figures 34–36.**
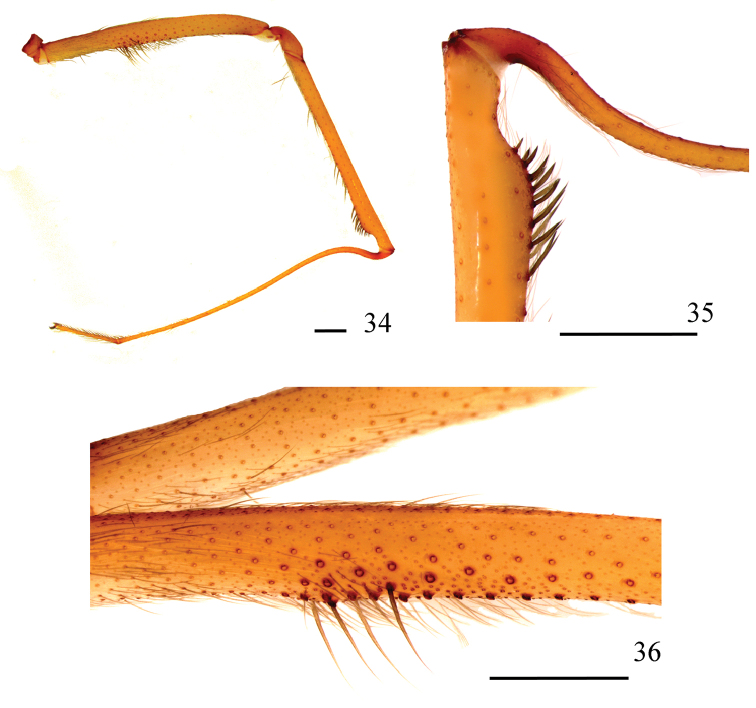
*Loxoscelescarinhanha* sp. n., holotype male (MZUSP 74436), left leg I **34** prolateral **35** detail of tibia and metatarsus joint, showing metatarsus curvature and macrosetae on distal tibia **36** detail of macrosetae on median portion of femur. Scale bar: 1 mm.

*Male holotype*: Total length 7.32. Carapace 3.63 long, 3.39 wide. Eye sizes and interdistances: ALE 0.22, PME 0.22, PLE 0.21, PME-PLE 0.05, PME-ALE 0.27; clypeus 0.38. Leg formula II, IV, III, I. Leg lengths: leg I: femur 7.18, patella 1.44, tibia 6.68, metatarsus 9.29, tarsus 2.18, total 26.77; II femur 9.69, patella 1.51, tibia 10.87, metatarsus 13.34, tarsus 2.23, total 37.64; III: 7.56, 1.33, 7.88, 9.97, 1.70, 28.44; IV: 8.37, 1.41, 8.54, 11.92, 2.16, 32.40. Palp: femur 1.92 long, 0.34 wide; patella 0.54 long, 0.41 wide; tibia 1.12 long, 0.57 wide; cymbium 0.61 long, 0.45 wide. Labium 0.89 long, 0.49 wide. Sternum 1.87 long, 1.74 wide. Femur I 1.9 times as long, tibia I 1.8 times as long and leg I 7.4 as long as carapace. Palpal femur 5.6 times longer than wide; tibia 2.0 times longer than wide; cymbium oval (Figs 32, 33). Bulb suboval and slightly larger than cymbium. Embolus thick and straight, with a curvature on apex, approximately 1.3 times longer than bulb length in retrolateral view, without carina (Figs 29–31). Femur I prolateral median area with a series of enlarged setae (Figs 34, 36). Metatarsus I strongly curved on its basal portion. Distal tibia I abruptly narrow, with a series of strong macrosetae before the constriction (Figs 34, 35). Cephalic region of carapace, fovea, and thoracic striae with long, greyish setae (Fig. 28). Carapace and chelicerae uniformly reddish (Fig. 28). Abdomen, legs, and palp light brown, covered by short, greyish setae. Coxae and sternum light brown; labium and endites slightly darker.

**Figures 37–41.**
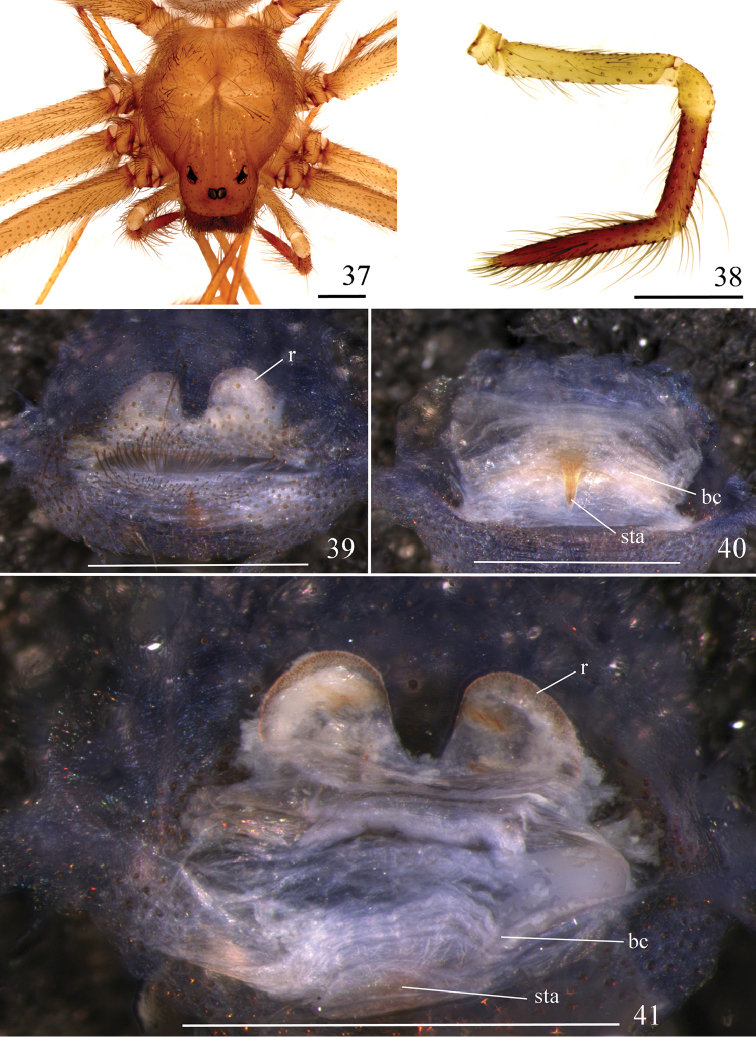
*Loxoscelescarinhanha* sp. n., paratype female (MZUSP 74437) **37** carapace and palp **38** left palp, prolateral **39−41** spermathecae **39** ventral **40** dorsal, with bursa copulatrix over receptacles **41** dorsal, bursa copulatrix unfolded below. Abbreviations: bc bursa copulatrix, r receptacle, sta sclerotized triangular area. Scale bar: 1 mm.

*Female paratype*: Total length 9.30. Carapace 3.99 long, 3.25 wide. Eye sizes and interdistances: ALE 0.20, PME 0.20, PLE 0.22, PME-PLE 0.05, PME-ALE 0.34; clypeus 0.41. Leg formula II, I, IV, III. Leg lengths: leg I: femur 6.79, patella 1.30, tibia 7.12, metatarsus 7.47, tarsus 1.82, total 24.50; II: 7.97, 1.40, 8.69, 9.30, 1.98, 29.34; III: 6.69, 1.29, 6.42, 7.48, 1.69, 23.57; IV: 7.23, 1.35, 7.20, 9.21, 1.69, 26.68. Palp: femur 1.61 long, 0.28 wide; patella 0.54 long, 0.34 wide; tibia 1.07 long, 0.26 wide; tarsus 1.67 long, 0.23 wide. Labium 0.67 long, 0.54 wide. Sternum 1.98 long, 1.68 wide. Femur I 1.7 times as long, tibia I 1.8 times as long and leg I 6.1 as long as carapace. Palpal femur 5.7 times longer than wide, tibia 4.1 longer than wide, tarsus not incrassate (Fig. 38). Spermathecae are a large, weakly sclerotized pouch with two large receptacles on its distal portion. Dorsal parts of bursa copulatrix have a small, sclerotized triangular area (Figs 39–41). Carapace with some sparse, long, greyish setae (Fig. 37). Carapace light brown, cephalic area, fovea, and border darker (Fig. 37). Chelicerae reddish brown. Abdomen greyish, legs light brown, both covered by short greyish setae. Palp femur and patella light brown, tibia and tarsus reddish brown (Fig. 38). Coxae and sternum light brown, labium and endites brown.

#### Etymology.

The specific name refers to the type locality of the species, Carinhanha, a municipality in the southwestern section of the state of Bahia, Brazil. The region possesses several cave systems with high diversity and a fragile subterranean fauna.

#### Remarks.

**Figures 42–47.**
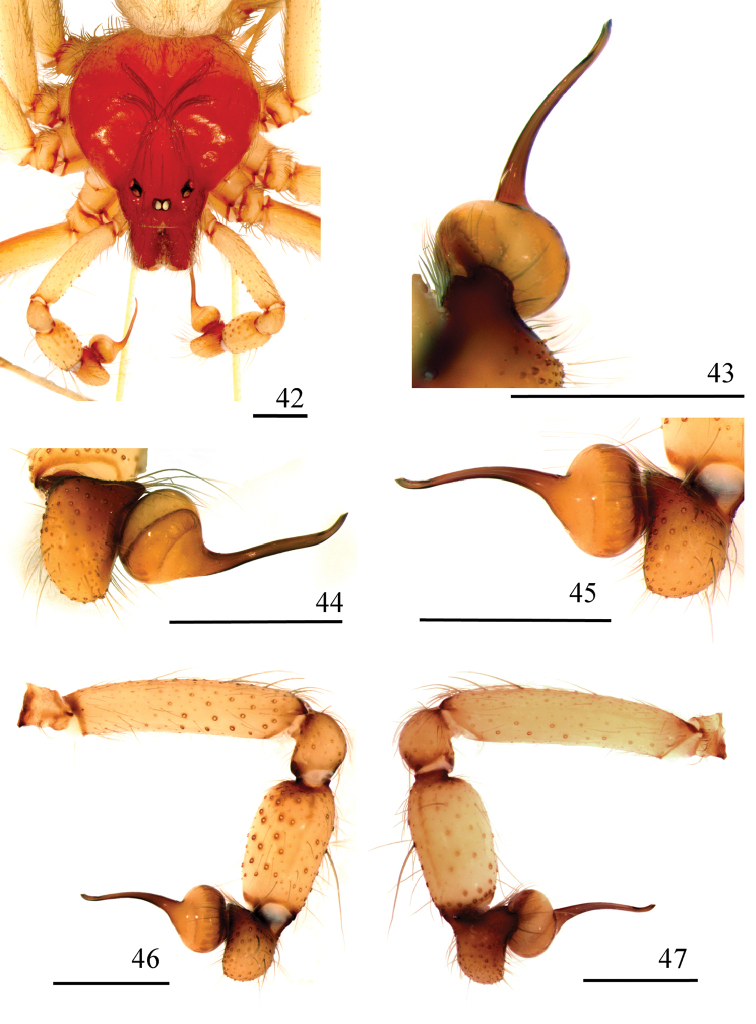
*Loxoscelescardosoi* sp. n., holotype male (MZUSP 74440) **42** carapace and palp **43−45** left palpal bulb **43** dorsal **44** prolateral **45** retrolateral **46, 47** left palp **46** prolateral **47** retrolateral. Scale bar: 1 mm.

**Figure 48.**
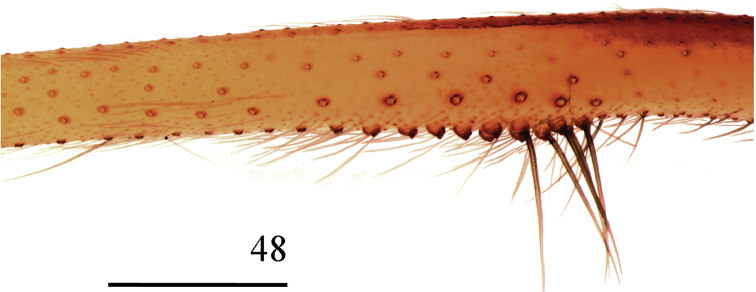
*Loxoscelescardosoi* sp. n., holotype male (MZUSP 74440), left leg I, detail of macrosetae on median portion of femur. Scale bar: 1 mm.

*Loxoscelescarinhanha* sp. n. and *L.cardosoi* sp. n. males have a uniformly reddish carapace (Figs 28, 42) instead of the brown marked carapace characteristic of the groups *gaucho* and *rufescens*/*amazonica*, and femur I has macrosetae on its prolateral median area (Figs 36, 48), which is exclusive of the two species. They occur in closer areas and are probably sister species. The inclusion of the two species in one of the groups defined by Gertsch (1967) for South American *Loxosceles* is not simple question. They could fit in either *gaucho* or *rufescens*/*amazonica* groups. Males of *gaucho* group have the cymbium and tibia subequal in length (Gertsch 1967). However, two species described more recently has slightly longer and slender tibia (*L.chapadensis* and *L.niedeguidonae*). Even though the tibia is not incrassate in these species, the cymbium is larger than the bulb, projecting forward. Considering the variation of tibia length and width in this group, we consider the cymbium size a better character to diagnose males of *gaucho* group. Males of the *rufescens*/*amazonica* group have the cymbium considerably shorter than tibia. More important, however, is they are never much more larger than the bulb. Based in this criterion, both *L.carinhanha* sp. n. and *L.cardosoi* sp. n. can be included in the *rufescens*/*amazonica* group (Figs 32, 33, and 46, 47). Concerning females, those of the *gaucho* group are readily recognizable by “the seminal receptacles attached to immovable, sclerotized, transverse plate” (Gertsch 1967). We noted that in species of *gaucho* group the receptacles are always slender and strongly sclerotized, except the apex and can be another diagnostic character. Those of the *rufescens*/*amazonica* group have the “seminal receptacles with a cluster of small, globular lobes at apex” (Gertsch 1967). More recently, at least two species were known to have a single large lobe at apex, *L.mahan* Planas & Ribera, 2015 from Canary Islands and *L.willianilsoni*, from Brazil (Fukushima et al. 2017). We consider that the main characters shared by females of *rufescens*/*amazonica* group is the spermathecae triangular shape, two free receptacles (not attached to a transverse sclerotized plate) with large basal transverse openings with or without sclerotized edges and two dark sclerotized lateral bands with distinct levels of sclerotization depending on the species (see Planas and Ribera 2015 and Fukushima et al. 2017 for spermathecae variation). *Loxoscelescardosoi* sp. n. females have a transverse sclerotized plate (compatible with those species of *gaucho* group) and the receptacles are short (contrary to *rufescens*/*amazonica* group) and broad (as in the *rufescens*/*amazonica* group). A single dark sclerotized band is present (another characteristic of *rufescens*/*amazonica* group). The bursa copulatrix is strongly sclerotized. The putative sister species, *L.carinhanha* sp. n. has spermathecae weakly sclerotized lacking a transverse sclerotized plate and the receptacles are free. The bursa copulatrix is weakly sclerotized, except for a central triangular area. In favor of the inclusion of *L.cardosoi* sp. n. and *L.carinhanha* sp. n. in *rufescens*/*amazonica* group are the short cymbium in males and the broad and no sclerotized receptacles in females. Additionaly, *L.carinhanha* sp. n. spermathecae have a single dark sclerotized band. There is no supporting character for the inclusion of males in the *gaucho* group. In females, *L.cardosoi* sp. n. has the characters transverse sclerotized plate and short receptacles, which are lacking in *L.carinhanha* sp. n. Therefore, it seems more parsimonious to include the two species in the *rufescens*/*amazonica* group, elevating to five the number of species of this group in South America. These two species are very distinctive of the other species of the group both in the New and the Old World.

It has been proposed the origin of *Loxoscelesrufescens* group in the Old World with a posterior introduction of *L.amazonica* during portuguese colonization of Brazil beginning in 1500 (Duncan et al. 2010). One of the evidences for the introduction hypothesis was the lack of other related species in South America (Duncan et al. 2010). Recently, Fukushima et al. (2017) described two species related with *L.amazonica* and *L.rufescens* from Brazil and argued contrary to this possibility for the short time (500 years) for speciation taking place. The discovery of two additional and very distinctive species reinforces the proposal of Fukushima et al. (2017). As the *Loxosceles* diversity in South America is still largely unknown, it is necessary more efforts to collect and describe species from more remote areas of Brazil, mainly those in the northeastern and central western regions, as the areas under study here, which seems to be a hot spot for *Loxosceles* diversity.

### Loxosceles cardosoi

Taxon classificationAnimaliaAraneaeSicariidae

Bertani, von Schimonsky & Gallão sp. n.

http://zoobank.org/FB08FAF6-0DFD-4E30-8C29-088423627E1F

Figs 1 , 42–47 , 48 , 49–54 , 56 , 57

#### Material examined.

Male holotype (MZUSP 74440) and female paratype (MZUSP 74441), 1 male, 4 females paratypes (MZUSP 74442), 2 males, 4 females paratypes (LES 14710), BRAZIL: *Bahia*, Carinhanha, Gruna da Altina cave (13°33'S, 43°45'W) 496 m a.s.l., M.E. Bichuette, N. Hattori and J.E. Gallão leg., 01.vi.2012.

#### Other material examined.

BRAZIL, *Bahia*: Carinhanha, Gruna da Altina Cave (13°33'S, 43°45'W) 496 m a.s.l., 10 immatures, M.E. Bichuette, N. Hattori and J.E. Gallão leg., 01.vi.2012 (LES 14711).

#### Diagnosis.

Males of *Loxoscelescardosoi* sp. n. resemble those of *L.carinhanha* sp. n. by having a group of macrosetae on the median prolateral area of femur I (Fig. 48). They can be distinguished from the males of *L.carinhanha* sp. n. by the slender embolus with a gentle curvature on its median area ending in a strong curvature on its apex (Figs 43–45) and the straight metatarsus I. Females of *L.cardosoi* sp. n. resemble those of *L.carinhanha* sp. n. by having spermathecae as a large, weakly sclerotized pouch with two large receptacles on its distal portion. Females of *L.cardosoi* sp. n. can be distinguished from those of *L.carinhanha* by the spermathecae having a sclerotized transverse plate, one dark sclerotized band reaching the basal area of each receptacle and dorsal and ventral parts of bursa copulatrix strongly sclerotized (Figs 51–54).

#### Description.

*Male holotype*: Total length 7.54. Carapace 3.70 long, 3.54 wide. Eye sizes and interdistances: ALE 0.23, PME 0.20, PLE 0.19, PME-PLE 0.02, PME-ALE 0.23; clypeus 0.30. Leg formula II, IV, III, I. Leg lengths: leg I: femur 7.39, patella 1.38, tibia 8.25, metatarsus 9.26, tarsus 1.77, total 28.05; II: 10.12, 1.43, 12.41, 13.65, 2.06, 39.67; III: 8.05, 1.36, 8.04, 9.82, 1.52, 28.79; IV: 8.67, 1.38, 9.04, 11.74, 2.03, 32.86. Palp: femur 2.10 long, 0.47 wide; patella 0.57 long, 0.51 wide; tibia 1.27 long, 0.67 wide; cymbium 0.72 long, 0.52 wide. Labium 0.89 long, 0.52 wide. Sternum 1.85 long, 1.82 wide. Femur I 2.0 times as long, tibia I 2.2 times as long, and leg I 7.6 as long as carapace. Palpal femur 4.5 times longer than wide, tibia 1.9 times longer than wide, cymbium oval (Figs 46, 47). Bulb suboval and approximately same size as cymbium. Embolus slender, with a gentle curvature to dorsal aspect on its middle and a strong curvature on apex, approximately two times longer than bulb length in retrolateral view, without carina (Figs 44, 45). Cephalic region of carapace, fovea and thoracic striae with long, greyish setae forming a pattern (Fig. 42). Carapace and chelicerae uniformly reddish (Fig. 42). Abdomen, legs and palp light brown, covered by short, greyish setae. Femur I dorsally brown on its base. Coxae and sternum light brown, labium and endites brown. Femur I prolateral median area with a series of macrosetae (Fig. 48).

**Figures 49–54.**
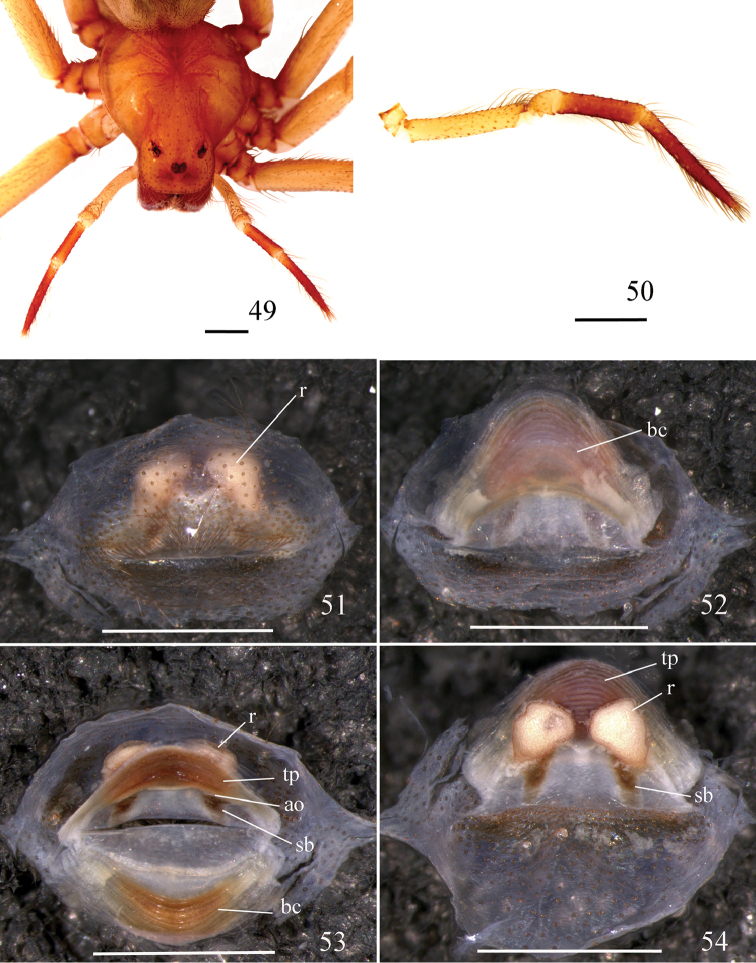
*Loxoscelescardosoi* sp. n., paratype female (MZUSP 74441) **49** carapace and palp **50** left palp, prolateral **51−54** spermathecae **51** ventral, cuticle not removed **52** dorsal, with bursa copulatrix over receptacles **53** dorsal, bursa copulatrix unfolded below **54** ventral, cuticle removed. Abbreviations: ao atriobursal orifice, bc bursa copulatrix, r receptacle, sb sclerotized bar, tp transverse sclerotized plate. Scale bar: 1 mm.

*Female paratype*: Total length 9.15. Carapace 4.05 long, 3.49 wide. Eye sizes and interdistances: ALE 0.26, PME 0.24, PLE 0.22, PME-PLE 0.04, PME-ALE 0.36; clypeus 0.49. Leg formula II, IV, I, III. Leg lengths (left): leg I: femur 7.45, patella 1.42, tibia 8.24, metatarsus 8.30, tarsus 1.69, total 27.10; II: 8.87, 1.48, 9.87, 10.22, 1.80, 32.24; III: 7.30, 1.48, 7.00, 8.17, 1.48, 25.43; IV: 8.10, 1.48, 8.20, 9.91, 1.73, 29.42. Palp: femur 1.36 long, 0.31 wide; patella 0.36 long, 0.41 wide; tibia 1.18 long, 0.29 wide; tarsus 1.78 long, 0.22 wide. Labium 0.66 long, 0.64 wide. Sternum 2.13 long, 1.79 wide. Femur I 1.8 times as long, tibia I 2.0 times as long, and leg I 6.7 as long as carapace. Palpal femur 4.4 times longer than wide, tibia 4.0 longer than wide, tarsus not incrassate (Fig. 50). Spermathecae are a large weakly sclerotized pouch with two large receptacles on its distal portion, a broad transverse sclerotized plate, and one dark sclerotized band reaching the basal area of each receptacle. Dorsal and ventral parts of bursa copulatrix strongly sclerotized (Figs 51–54). Cephalic region of carapace, fovea, and thoracic striae with long, greyish setae (Fig. 49). Carapace light brown, cephalic area slightly darker (Fig. 49). Chelicerae brown. Abdomen and legs light brown, covered by short, greyish setae. Palp femur and patella light brown, tibia and tarsus reddish brown (Fig. 50). Coxae and sternum light brown; labium and endites brown.

#### Etymology.

The specific name is in honor of Dr. João Luiz Costa Cardoso, a physician who worked for several years at the Hospital Vital Brazil, Instituto Butantan, São Paulo, Brazil, treating bites and stings of venomous animals and publishing several related articles.

#### Remarks.

See remarks under *L.carinhanha* sp. n.

**Figures 55–57.**
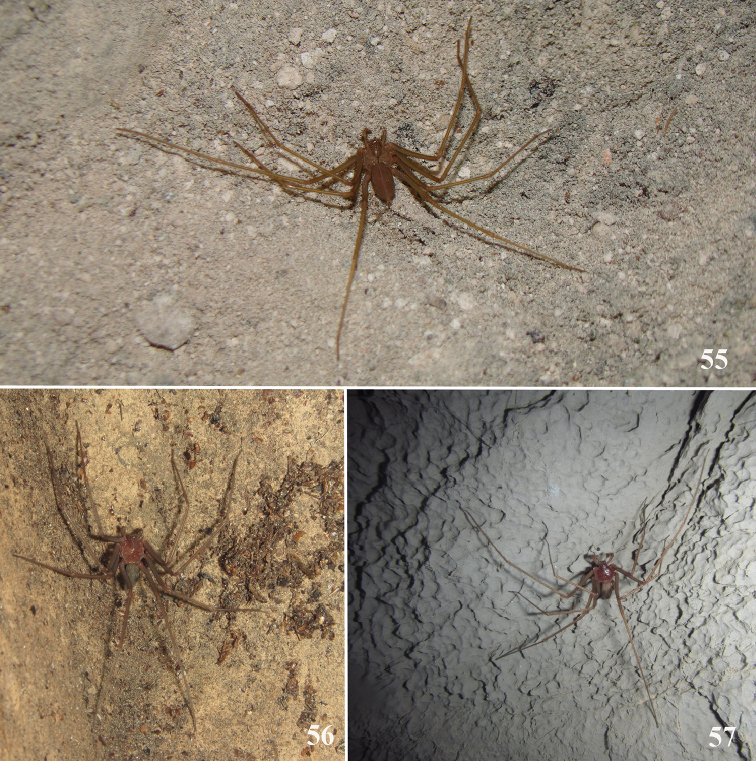
Living specimens in their habitats. **55***Loxoscelesericsoni* sp. n. female, Bonita Cave, Peruaçu Caves National Park, Januária, state of Minas Gerais, Brazil **56, 57***Loxoscelescardosoi*, Gruna da Altina Cave, Serra do Ramalho karst area, Carinhanha, state of Bahia, Brazil. **56** Female **57** Male. Photographs by PP Rizzato (**55**), ME Bichuette (**56, 57**).

## Discussion

According to Trajano and Carvalho (2017), troglophilic populations are easily found more in subterranean habitats than in epigean habitats, probably by the differences in species dynamics. Generally, troglophiles present higher densities in subterranean habitats and low densities on the surface and they can move between them (Trajano and Carvalho 2017). The presence of individuals at all ages in subterranean habitats is one of the strongest pieces of evidence for troglophilic populations, contemplating such distribution along the years including different annual cycles.

Autapomorphic states, known as troglomorphisms in troglobitic species, evolving because the subterranean habitats (by natural selection, neutral mutation or even pleyotropy) are relevant clues to state if a species are obligatory and exclusive cave-dwelling, however, this method is only valid when used in a comparative method.

*Loxosceleskarstica* sp. n., *L.carinhanha* sp. n. and *L.cardosoi* sp. n., only found inside caves, do not show any troglomorphisms, at least morphological, such as elongated appendices, reduction of visual organs, sclerotization degree or pigmentation, when compared with other *Loxosceles* species, as observed in *L.troglobia*, the only troglobitic representative of *Loxosceles* in Brazil (Souza and Ferreira 2018). It is noteworthy that epigean habitats for these species are very dry, mainly in the winter season in Brazil, which may lead not to find these species in the surface, however, individuals of this new *Loxosceles* species may be encountered in suitable habitats on epigean surface. Presence of troglomorphisms do not define troglobitic species, but in most cases are cause of the incompatibility of troglobitic species living in epigean habitats (Trajano and Carvalho 2017), as seen in *L.troglobia*.

The presence of three new species (*L.ericsoni* sp. n., *L.carinhanha* sp. n., and *L.cardosoi* sp. n.) with very distinct morphological characteristics in a relatively small area (Fig. 1) indicates that the regions of Peruaçu and Serra do Ramalho are important centers for *Loxosceles* distribution, which remains poorly studied.

The karst areas of Peruaçu and Serra do Ramalho have different conservation statuses. Peruaçu’s caves are under legal protection as part of a National Park (Peruaçu Caves National Park-PCNP), and part of its cave fauna is included in the Brazilian RedList (at present, four species); by contrast, the Serra do Ramalho karst area has no legal protection, and the main strategy to protect its karst, caves, and cave fauna is to use the Brazilian RedList, since six of the 14 troglobites are included on this list (Gallão and Bichuette 2018). Both areas are included in the governmental program for priority areas for conservation (PAN), but until now, no action has been proposed. The main threats to both regions are deforestation in Itacarambi and Januária and deforestation together with mining projects in the Serra do Ramalho karst area (Gallão and Bichuette 2018). Although the new species of *Loxosceles* described herein are not obligatorily cave-dwelling, they are endemic to two regions with a high degree of endemism of troglobites and are part of a fragile community, representing a strong argument for protection of these regions’ cave fauna.

## Supplementary Material

XML Treatment for Loxosceles ericsoni

XML Treatment for Loxosceles karstica

XML Treatment for Loxosceles carinhanha

XML Treatment for Loxosceles cardosoi
